# Current Transients in Graphene Electronics under Single‐Particle Irradiation

**DOI:** 10.1002/smsc.202300011

**Published:** 2023-06-11

**Authors:** Wanzhen He, Linxin Zhai, Chi-Yung Yam, Zhiping Xu

**Affiliations:** ^1^ Applied Mechanics Laboratory Department of Engineering Mechanics Tsinghua University Beijing 100084 China; ^2^ Shenzhen Institute for Advanced Study University of Electronic Science and Technology of China Shenzhen 518000 China

**Keywords:** graphene electronics, open systems, peak transient current, real-time time-dependent density-functional theory, single-particle irradiation

## Abstract

Low‐dimensional materials hold great promise in next‐generation electronics. However, the performance of such devices is susceptible to external perturbations such as irradiation due to the high exposure of constituent atoms to the environment. Herein, real‐time time‐dependent density‐functional theory at the tight‐binding level extended to open systems for electrons and Ehrenfest dynamics for ions is developed and used to explore the effects of single‐H irradiation on graphene electronics. The results show that the peak current displays distinct energy and site dependences, which are largely different from the dependences of the stopping powers. Charge‐density analysis shows that the current transients are driven by delocalized plasmonic excitation, in contrast to localized electronic excitation, which plays a crucial role in the stopping power. The site dependence of the transient current is determined by the electron density at the irradiation site and the ionic charges. These findings highlight the roles of lattice discreteness and electronic structures of materials, which have been overlooked in previous studies based on theoretical formulations and semiempirical models. Using the insights gained from the calculations and the dataset constructed under typical space‐irradiation conditions, the device responses of graphene nanoelectronics are modeled, laying the ground for device design in the space environment.

## Graphical abstract

Real‐time dynamics of electronic excitation in graphene transistors is explored by quantum simulations. The open‐system approach resolves current transients resulting from single‐particle irradiation events, showing strong patterns of energy and site dependence. It is suggested in the results that transient current and stopping powers should be considered as two separate risk‐assessment indicators for nanoelectronics operating under irradiation conditions.

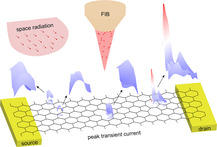

## 1 Introduction

Single‐particle irradiation (SPI) is the stepping stone to the understanding of particle–matter interactions and has significant implications for scientific and technological advances in space science, material modification, imaging, and radiation therapy.^[^ ^1^, ^2^, ^3^, ^4^ ^]^ Electronic or electromechanical devices equipped in space missions experience single‐event effects (SEEs) caused by cosmic rays or energetic protons. Soft errors such as single‐event upset (SEU) and hard errors such as single‐event latch‐up (SEL) are exemplary radiation events, which can modify the working states of devices.^[^ ^2^ ^]^ Recent proton radiation experiments on carbon nanotube (CNT) field‐effect transistors (FETs) show major and minor SEE signals of approximately 100 nA.^[^ ^5^ ^]^ Compared to conventional silicon‐based radiation‐hardened integrated circuits (ICs), comprehensive radiation damage tolerance was also observed in CNT electronics such as FETs and static random‐access memories (SRAMs).^[^ ^6^ ^]^ However, the state‐of‐the‐art understanding of SEEs is limited to the device performance and relies heavily on semiempirical models.^[^ ^7^ ^]^ The underlying microscopic processes of lattice and electronic excitation were not considered. Quantitative measures of the SPI effect are thus mainly on the stopping power and linear transfer energy (LET), while temporal changes in device performance such as the operating current cannot be assessed.^[^ ^8^, ^9^ ^]^

2D electronics are renowned for their exceptional performance.^[^ ^10^ ^]^ However, they are susceptible to radiation due to the extremely large surface‐to‐volume ratios.^[^ ^11^, ^12^, ^13^, ^14^ ^]^ Empirical or first‐principles molecular dynamics (MD) simulations with electrons in their ground states were employed to investigate the processes of ion stopping and defect production. These processes are controlled by various factors such as the types of projectiles and their momentum, their interaction with the substrate, and the sites of irradiation. Real‐time time‐dependent density‐functional theory (RT‐TDDFT) was developed to capture electronic excitation^[^ ^15^, ^16^, ^17^, ^18^, ^19^ ^]^ and reveal the exchange and deposition pathways of both charge and energy. These studies are limited to closed systems in previous studies, and the open nature of electron flow from (to) the source (drain) in the device has not been explored. The working state indicators of electronic devices, such as the transient current under irradiation, remain largely unexplored, making it challenging to design electronic and electromechanical devices that are able to operate reliably in harsh environments such as the space.

In this study, we develop a first‐principles framework based on the linear‐scaling quantum chemistry program based on the localized‐density‐matrix (LDM) method, LODESTAR.^[^ ^20^ ^]^ We combine the real‐time time‐dependent density‐functional tight‐binding (RT‐TDDFTB) models with the nonequilibrium Green function (NEGF) formalism^[^ ^21^ ^]^ to analyze the open systems of electrons (TDDFTB‐OS).^[^ ^20^, ^22^ ^]^ The SPI effects in graphene electronics are explored by introducing a coupling to the Ehrenfest dynamics of the ions and irradiation projectiles. The device responses are measured by the current transients and the stopping powers. The energy and site dependence of these responses as well as the roles of electron excitation and nuclear displacement are analyzed and discussed.

## 2 Methods

**Figure 1:**
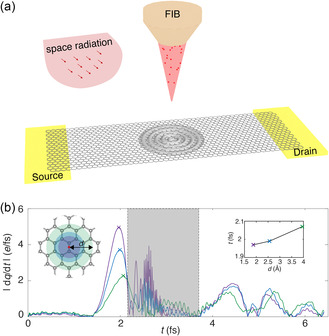
Electronic excitation in graphene electronics under single‐particle irradiation (SPI). a) Schematic illustration of charge‐density waves in a graphene device under SPI, from space radiation or the focused ion beams (FIBs), for example. b) The rate of change in the charge enclosed by a cutoff distance (*d*) from the site of single‐H irradiation (the red dot), |dq/dt|. The atoms in the device region are fixed (the constrained model) for the results plotted in this panel (see Figure S2, Supporting Information, for free‐standing models). The time window for H to pass through the graphene sheet within a thickness of 0.2 nm is marked by a gray background. The relation between the time of occurrence *t* of $|dq(t)/dt{|}_{max}$ and the cutoff distance *d* is plotted in the inset. The wave speed $v=1.98\times 10^{6}\;m\;s^{-1}$ is close to the Fermi velocity in graphene, $v_{F}=3\times 10^{6}m\;s^{-1}$.^[^ ^29^ ^]^

In our TDDFTB‐OS approach, the electron density in a subsystem of interest determined that of the entire system through analytical continuation. This important assertion was proved by the holographic electron‐density theorem,^[^ ^23^, ^24^ ^]^ and extended to solve time‐dependent problems.^[^ ^25^ ^]^ For a typical open electronic system (**Figure** **1**a and S1, Supporting Information), the time evolution of reduced single‐electron‐density matrix (RSDM) of the device region, $\sigma_{D}(t)$, can be described by the Liouville–von Neumann equations

(1) $$i\frac{d}{dt}\sigma_{D}(t)=[h_{D}(t),\sigma_{D}(t)]-i\sum_{\alpha =\text{L,R}}Q_{\alpha }(t)$$

where $h_{D}(t)$ is the Kohn–Sham Fock matrix of the device region described at the density functional based tight binding (DFTB) level.^[^ ^26^ ^]^ This approach offered high computational efficiency to handle large systems consisting of hundreds of atoms using the LODESTAR code. $Q_{\alpha }(t)$ is the dissipative term resulting from the interaction with the left (L) and right (R) leads. The time‐dependent electric current through the electrode *α* is

(2) $$J_{\alpha }(t)=-e\text{Tr}[Q_{\alpha }(t)]$$

where $Q_{\alpha }(t)$ is evaluated through the NEGF formalism. We employed the adiabatic wideband limit approximation for the electrodes,^[^ ^27^ ^]^ which assumed that the bandwidths of electrodes were infinitely large, and their line‐widths ($\Delta_{\alpha }(t)$) were independent on the energy of electrons. The adiabatic approximation for the memory effects was introduced to evaluate the time integral of $h_{D}(t)$ and $\Delta_{\alpha }(t)$. In this way, $Q_{\alpha }(t)$ was simplified into

(3) $$Q_{\alpha }^{\text{AWBL}}(t)=\{{\tilde{\Lambda }}_{\alpha },\sigma_{D}\}+P_{\alpha }(t)+{\left[P_{\alpha }(t)\right]}^{†}$$

where ${\tilde{\Lambda }}_{\alpha }$ is the imaginary part of the self‐energy evaluated at the Fermi energy for the electrode *α*, and

(4) $$\begin{array}{c} P_{\alpha }(t)≃-\frac{i}{\pi }\{U_{\alpha }^{+}(t)\int_{-\infty }^{+\infty }d\varepsilon f_{\alpha }(\varepsilon )e^{i\varepsilon t}\\ \times [\frac{1}{\varepsilon -h_{D}(0)+i\tilde{\Lambda }}-\frac{1}{\varepsilon -h_{D}(t)+i\tilde{\Lambda }+\Delta_{\alpha }(t)}]\\ +\int_{-\infty }^{+\infty }\frac{f_{\alpha }(\varepsilon )}{\varepsilon -h_{D}(t)+i\tilde{\Lambda }}d\varepsilon \}{\tilde{\Lambda }}_{\alpha } \end{array}$$

(5) $$i{\dot{U}}_{\alpha }^{+}(t)=[h_{D}(t)-i\tilde{\Lambda }-\Delta_{\alpha }(t)]U_{\alpha }^{+}(t)$$

where $U_{\alpha }{\left(t\right)}^{+}$ and $f_{\alpha }(\varepsilon )$ are the propagator of electrons and the Fermi–Dirac distribution function, respectively.

The electron dynamics was coupled to nuclear motion, and the nonadiabatic effects were taken into account via the Ehrenfest dynamics.^[^ ^20^ ^]^ The corresponding equation of motion for the nuclei is given by

(6) $$\begin{array}{c} F_{A}(t)=-\text{Tr}\left[\frac{\partial h_{D}^{0}}{\partial R_{A}}\sigma_{D}\right]-\sum_{B\in D}\sum_{\nu \in B}\sum_{\mu \in A}{\left(\sigma_{D}\right)}_{\mu \nu }B_{\mu \nu }^{A}\cdot \sum_{C}(\gamma_{\text{AC}}+\gamma_{\text{BC}})\Delta q_{C}\\ -\Delta q_{A}\sum_{B\in D}\frac{\partial \gamma_{\text{AB}}}{\partial R_{A}}\Delta q_{B}-\sum_{B\in D,B\neq A}\frac{\partial E_{\text{rep}}(\left|R_{\text{AB}}\right|)}{\partial R_{A}}\\ +\text{Tr}\left[h_{D}\sigma_{D}{\left(B^{A}\right)}^{†}S_{D}^{-1}+h\text{.c}\text{.}\right]+\sum_{\alpha =\text{L,R}}\left[h_{D\alpha }\sigma_{\alpha D}{\left(B^{A}\right)}^{†}S_{D}^{-1}+h\text{.c}\text{.}\right] \end{array}$$

where A, B, and C are the indices of atoms, *μ* and *ν* are the indices of atomic orbitals (AOs), and h.c. is the Hermite conjugate. **R**_A_ and **F**_A_ are the displacement of atom A and the force exerted on it, respectively. $h_{D}^{0}$ is the Fock matrix of the neutral DFTB system. $B_{\mu \nu }^{A}=\left\langle \phi_{\nu }|\frac{\partial \phi_{\mu }}{\partial R_{A}}\right\rangle$ is the partial derivative of the overlap matrix $S_{D}$ with respect to the nuclear displacement $R_{A}$, and $\phi_{\mu }$ is the basis function of AO *μ*. $\Delta q_{A}$ is the induced Mulliken charge on atom A. $\gamma_{\text{AB}}$ describes the interaction between the induced charges located on atoms A and B, and $E_{\text{rep}}$ gives the pair‐wised repulsive energy between the two atoms.^[^ ^26^ ^]^ Equations (1) and (6) govern the time evolution of electrons and nuclei in the system, respectively. The time‐dependent RSDM, $\sigma_{D}(t)$, contained all electronic information of the simulation region, which allowed us to analyze the evolution of charge densities in the system.

In addition to the RT‐TDDFTB calculations using our LODESTAR code, we also performed closed‐system RT‐TDDFT calculations using the *Spanish Initiative for Electronic Simulations with Thousands of Atoms* (SIESTA) code^[^ ^28^ ^]^ to investigate the site dependence of SPI (Supplementary Note 1, Supporting Information).

## 3 Results and Discussion

The model device used in this study follows a lead‐scatter‐lead setup of an H‐terminated zigzag‐edged graphene nanoribbon (ZGNR) (Figure 1a and S1a, Supporting Information). ZGNRs are chosen because they are metallic and feature unique topological edge states.^[^ ^30^ ^]^ The dimensions of the device region (length L=2.0 nm, width W=1.4 nm, 112 C atoms in total) are in accordance with the recent experimental work on graphene FETs.^[^ ^31^ ^]^ Devices of armchair‐edged graphene nanoribbons (AGNRs) with width‐dependent bandgaps are also explored for comparison. An H atom is launched at a normal angle of incidence from a distance of 0.4 nm away from the basal plane of graphene. The mass and velocity of the projectile are with a mass of $m_{H}$ and $v_{H}$, respectively, and the kinetic or irradiation energy is set to $K=\frac{1}{2}m_{H}v_{H}^{2}=10\text{ to }10^{7}$ eV. This range is chosen to include the characteristic energy scales of nuclear and electronic stopping. For comparison, the typical ranges of kinetic energies carried by the ions are 10–50 keV for focused ion beams (FIBs)^[^ ^32^ ^]^ and keV to TeV for space radiation,^[^ ^33^ ^]^ respectively. Furthermore, we will show in this work that the SPI effects are no longer significant above 10 MeV. The atoms in the system are either fixed (“constrained,” e.g., by the substrate) or free to move (“free‐standing”) to explore the interplay between electronic excitation and nuclear displacement. To ensure the convergence of simulation results, time steps for integrating the RT‐TDDFTB equations are selected adaptively between 0.1 and 5 as. The structures are relaxed prior to the transport calculations. The bias voltage is set to $V_{b}=0.1\;V$, which is of the same magnitude as that applied in experiments^[^ ^5^ ^]^ and previous RT‐TDDFT simulations.^[^ ^22^ ^]^

We begin our analysis by examining the evolution of atomic charges (*q*) within a distance of *d* from the irradiation site (e.g., the bond centers in graphene) under 100 eV H irradiation (Figure 1b and S2, Supporting Information). As the H atom passes through graphene within a space interval of 0.2 nm, high‐frequency changes in the charge (dq/dt) are induced. The time evolution of |dq(t)/dt| after the ion leaves graphene shows damped oscillation, which is consistent with our previous RT‐TDDFT study in the closed‐system approach.^[^ ^16^ ^]^ The first peak in the time evolution of |dq(t)/dt| features the maximum amplitude and is identified as $|dq(t)/dt{|}_{\text{max}}$, the onset of which is postponed as *d* increases. This observation suggests that the SPI effect perturbs the charge‐density distribution and results in subsequent outward propagation of charge‐density waves from the irradiation site. The wave speed $v=1.98\times 10^{6}m\;s^{-1}$ is close to the Fermi velocity of graphene ($v_{F}=3\times 10^{6}m\;s^{-1}$ ^[^ ^29^ ^]^), but one order of magnitude higher than the speed of ionic motion ($v_{H}$). The rate of change in the charge displays superposed features of short‐ and long‐time oscillations, with scales defined by the lattice constant and the size of the device, respectively. This feature is enhanced in the free‐standing model, where the atomic displacement is not constrained (Figure S2, Supporting Information). Due to the excited‐state nature of electrons, the Born–Oppenheimer approximation fails, and a TDDFTB‐OS scheme should be developed to explore the transient responses of electronic devices under irradiation.

Energy deposition under SPI is quantified through the stopping power *S*, which can be measured experimentally^[^ ^6^, ^34^ ^]^ or predicted by RT‐TDDFT calculations that reveal electron capture and emission processes.^[^ ^17^, ^19^, ^35^ ^]^ The stopping power S(K) shows two peaks at irradiation energies of about K≈10 eV and ≈100 keV, which corresponds to nuclear stopping with minor plasmonic electronic excitation^[^ ^16^, ^17^ ^]^ and the dominance of electronic stopping,^[^ ^36^ ^]^ respectively. Charge‐density analysis reveals delocalized plasmonic excitation at a characteristic energy scale of ≈15.3 eV, which agrees with the energy of the σ+π plasmon (≈15 eV) measured in electron energy loss spectroscopy (EELS),^[^ ^37^ ^]^ as well as theoretical predictions (≈15.4 eV) from DFT calculations with local field correction.^[^ ^16^ ^]^

In an open system with electronic transport, the effect of SPI can be assessed by the shift in the current–voltage (*I–V*) curve^[^ ^38^ ^]^ or the charge collected at terminals that should be maintained below a critical value of $\left(q_{\text{cr}}\right)$ to prevent device failure.^[^ ^2^ ^]^ Our simulation results reveal that SPI‐induced plasmonic excitation exhibits wavy features and the charge exchange is a more significant process than charge deposition (Figure 1 and S2, Supporting Information). Therefore, our subsequent discussion focuses on the transient current, a crucial quantity for measuring device performance.

**Figure 2:**
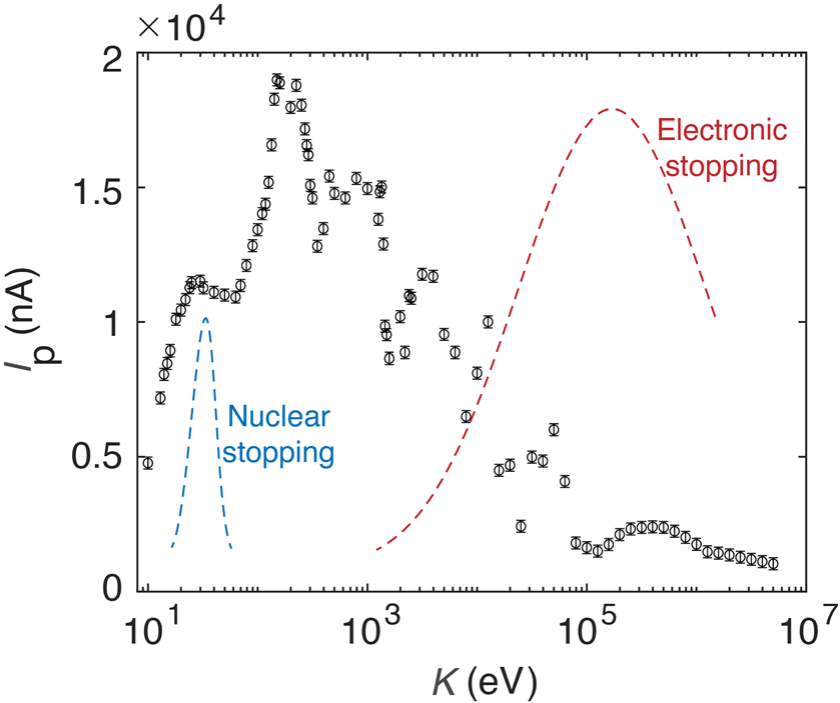
Peak transient current under single‐H irradiation on the bond centers of graphene. The steady‐state current in absence of SPI is *I*_0_ = 350 nA. The value of peak current *I*_P_ is evaluated with *K* ranging from 10 to 10 MeV. The dashed curves show the nuclei and electronic stopping powers S(K). The peaks of S(K) are located at K≈10 eV and ≈100 keV, respectively, and their amplitudes are only for illustration.

We choose the amplitude of the peak transient current (*I*_P_) under SPI as the primary risk‐assessment indicator for graphene electronics (**Figure** **2**). The steady‐state current calculated in the absence of SPI is *I*_0_ = 350 nA, which is comparable to the reported value for a graphene nanoribbon (GNR) FET under the same bias voltage.^[^ ^22^ ^]^ The values of *I*_P_ are calculated for H irradiation on the bond‐center sites, with *K* ranging from 10 to 10 MeV. Energy scales within this range are sufficiently low to exclude the relativistic effect but high enough to modify the working state of electronic devices, even at a relatively low value of K=10 eV, where the projectile is reflected. The device responses in $I_{p}(K)$ measured from the TDDFTB‐OS simulations show a shoulder‐peak feature and irregularly declining patterns for *K* ranging from 150 eV to 100 keV. This feature is very different from that of S(K) and can be attributed to the fact that the peak energy K=150 eV is closely tied to plasmonic excitation in graphene.^[^ ^16^ ^]^ For K<150 eV, the charge exchange and transfer are limited, while for K>150 eV, the timescale for H–matter interaction (τ≲1 fs for H traveling for 0.2 nm) is too short to activate the exchange of electrons through delocalized plasmonic electronic excitation (τ≈0.3 fs). For K≳100 keV, SPI cannot change the charge state of the projectile (Figure S3, Supporting Information). Despite being weak, the two characteristic peaks present in S(K) are also observable in the corresponding *K* range for $I_{p}(K)$. The lower amplitude of the peak current in the MeV range for $I_{p}(K)$, similar to that in S(K), indicates that localized charge excitation contributes to the stopping power but has little effect on the transient current. Therefore, *I*_P_ and *S* are independent measures of the irradiation effect and should be evaluated to assess soft errors and hard errors, respectively.

**Figure 3:**
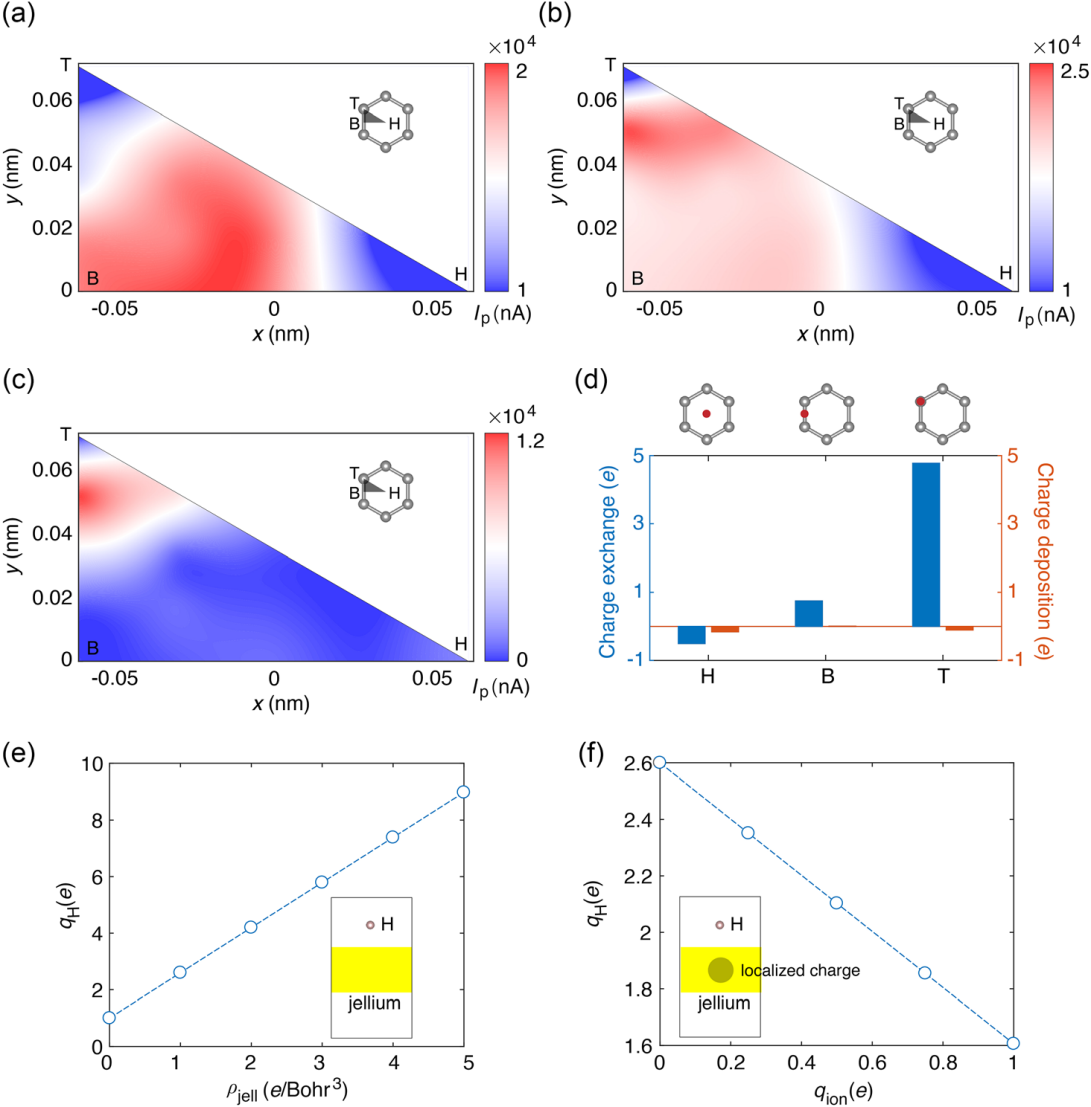
Site dependence of the peak current in graphene. a,b) 2D *I*_P_ map for irradiation sites sampled in a representative triangle of graphene, which is calculated at K=150 eV and $V_{b}=0.1\;V$. The atoms in the device region are set to be fixed or free to move in (a) and (b), respectively. c) Difference between the 2D *I*_P_ maps in free‐standing and constrained devices. d) Real‐time time‐dependent density‐functional theory (RT‐TDDFT) calculations of charge exchange and deposition for single‐H irradiation on the center of the hexagon (H), bond center (B), and the top of atoms (T) in the closed‐system approach (Supplementary Note 1, Supporting Information). e) Dependence of the atomic charge of hydrogen ($q_{H}$) on the electron density ($\rho_{\text{jell}}$) in the jellium slab. f) Dependence of $q_{H}$ on the charge of ion ($q_{\text{ion}}$) modeled by confining the positive charge in a localized sphere with $\rho_{\text{jell}}$ = 1 *e* Bohr^−3^.

The stopping power of materials under irradiation is typically modeled using atomic core and bond‐based semiempirical formulas that require only atomic composition information. The electronic structures of materials are not explicitly included, for example, by Ziegler et al.^[^ ^34^ ^]^ This overlooked site dependence of irradiation effects is resolved here by RT‐TDDFT simulations, the results of which show that energy transfer is positively correlated to the local charge density.^[^ ^19^ ^]^ We explore the site dependence of *I*_P_ by creating a 2D map in a reduced, triangular region following the lattice symmetry (**Figure** **3**a). A uniform grid of sampling is created to calculate *I*_P_ for site‐specific ion‐irradiation simulations with identical parameters (Figure S4, Supporting Information), and the values of *I*_P_ are interpolated over the whole region via bi‐harmonic splines. For our discussion here, we choose K=150 eV, which corresponds to the most significant response in *I*_P_. To clarify the relationship between the site‐dependent values of *I*_P_ and the electronic structures of materials, we explore the effects of electron density and ionic charges using a jellium–slab model with uniformly distributed electrons^[^ ^39^ ^]^ as implemented in the GPAW code.^[^ ^40^ ^]^ These results suggest that a higher charge density leads to a more significant accumulation of electrons on H (Figure 3e). Meanwhile, the presence of ionic charges (implemented through a confining potential with the same total number of electrons in the slab, Figure 3f) suppresses the responses due to the Coulombic attraction with the electrons. As a result, *I*_P_ is low at the H site with low electron density and at the T site in the presence of a positive C ion (Figure 3a). In comparison, the site dependence of the stopping power originates from the spatial distribution of the charge density,^[^ ^41^ ^]^ while that of transient current could be modified by the ionic charges.

The statistics indicate that the induced current values are in the range between $0.51\times 10^{4}$ and $1.98\times 10^{4}\;\text{nA}$ for the constrained device (Figure 3a) and between $0.47\times 10^{4}$ and $2.40\times 10^{4}\;\text{nA}$ for the free‐standing device (Figure 3b). The difference in their *I*_P_ maps identifies the effects of nuclear displacement (Figure 3c). In the constrained model, the responses measured in *I*_P_ are ranked from largest to smallest at the bond‐center (B), top‐of‐atom (T), and center‐of‐hexagon (H) sites, respectively. Though lattice excitation generally lags behind electronic processes and contributes less to the perturbation on the current in the range of high *K* values under consideration, irradiation on sites close to the nucleus can induce its displacement which may modify device performance. Consequently, irradiation near the T site elevates *I*_P_ in the free‐standing device (Figure 3c). This excitation of nuclear displacement also explains the strong charge exchange near the T site in the closed‐systems approach^[^ ^16^, ^17^, ^18^, ^42^ ^]^ (Figure 3d).

**Figure 4:**
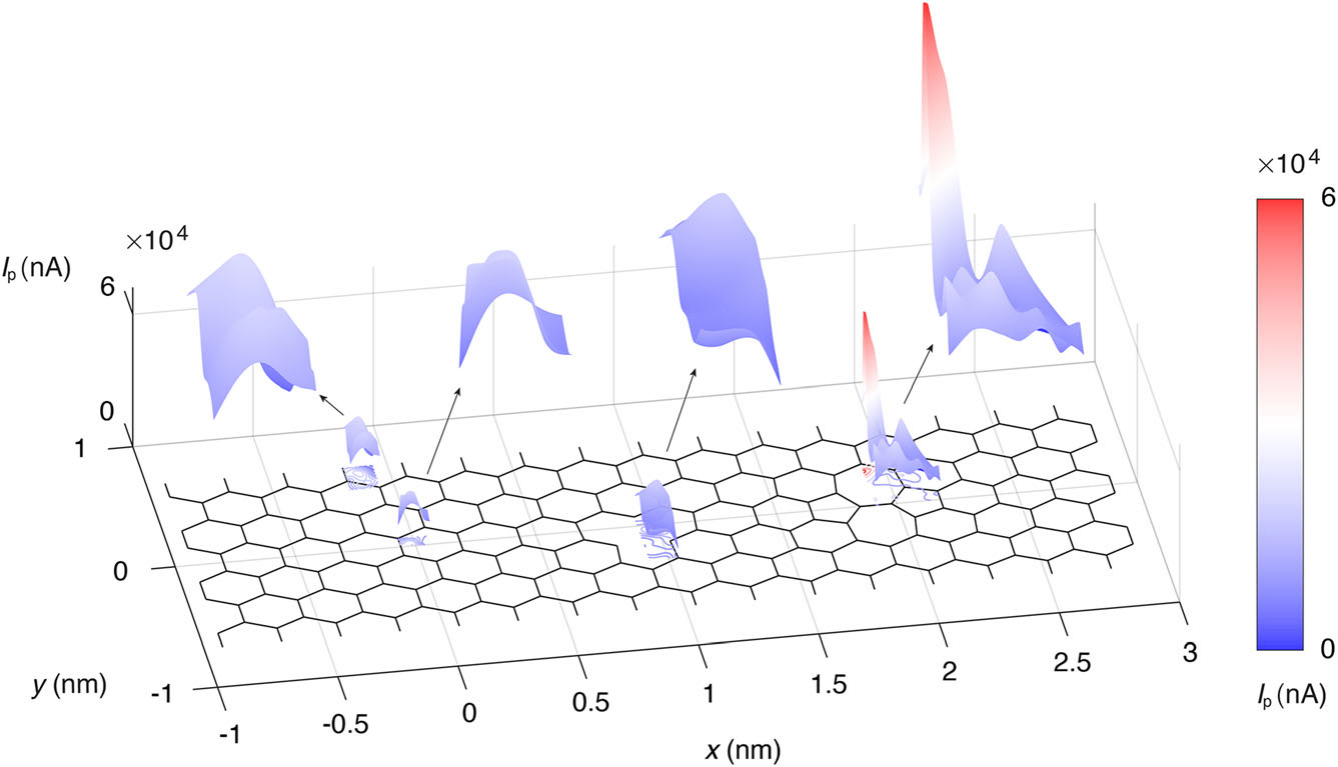
Contours of the peak transient current on pristine and defective sites in graphene. Contours of *I*_P_ in regions of the edge, pristine, single vacancy, and Stone–Wales defects (from left to right), interpolated from sampled sites in Figure S4, Supporting Information. The irradiation energy *K* is 150 eV, and the bias voltage is *V*_b_ = 0.1 V. The atoms in the device region are fixed.

In graphene electronics, interior and edge defects have a strong impact on the electronic transport processes and optoelectronic responses.^[^ ^43^, ^44^ ^]^ The SPI process is sensitive to these defects, which would then modulate the device response in the transient current. The *I*_P_ map at edges and defect sites (e.g., vacancies and Stone–Wales defects) are summarized in **Figure** **4** and S5, Supporting Information. The strong responses at the edges or vacancies can be attributed to the reduced effects from the C ions, while for Stone–Wales defects, multiple peaks are identified due to the distorted electronic structures. The responses in free‐standing models are stronger than those in the constrained model. Therefore, *I*_P_ in the graphene device is affected by both electronic and lattice structures under SPI. Simulations are also performed for the AGNR devices, the results of which confirm the roles of both the charge density and ionic charges in modulating the transient current (Figure S6, Supporting Information). However, the responses are much weaker due to the lower conductance compared to the ZGNRs (Figure S7, Supporting Information).

**Figure 5:**
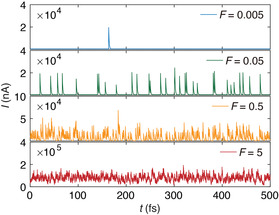
Time‐dependent signals in a graphene device under different irradiation fluxes. Particle irradiation events are considered to be uncorrelated with a damping period of 2 fs based on our time‐dependent density‐functional tight‐binding open systems of electrons (TDDFTB‐OS) simulations. The probability of the single irradiation event is determined by the flux *F*, which is chosen to be: a) 0.005, b) 0.05, c) 0.5, and d) 5 counts fs^−1^. The transient current is calculated from the TDDFTB‐OS simulation results sampled in the pristine graphene lattice (Figure 4). The irradiation energy is *K* = 150 eV, and the current in absence of SPI is 350 nA under a bias voltage of *V*_b_ = 0.1 V.

The responses of a ZGNR device can be constructed from our simulation results (Figure S8, Supporting Information). We develop a Monte Carlo (MC) algorithm (Supplementary Note 2 and Figure S9, Supporting Information) to simulate the time series of current signals under an irradiation flux *F* (**Figure** **5**). We assume that the SPI events are independent, which is reasonable given the short damping timescale of $\tau_{d}\approx 2\text{fs}$ according to our simulation results. The output signal is superposed from the irradiation events. In the space, solar winds can be intensive and yield a high proton flux of 10^8^ particles cm^−2^ s^−1^.^[^ ^45^ ^]^ Proton testing in electronics radiation characterization for space applications typically has a flux ranging from 10^4^ to 10^10^ particles cm^−2^ s^−1^,^[^ ^46^ ^]^ which corresponds to $F{=10}^{-21}\text{ to }10^{-15}$ counts fs^−1^ for a 10^4^ nm^2^ area. In commercial FIBs used for defect implantation^[^ ^47^, ^48^ ^]^ and microstructure modification,^[^ ^49^ ^]^ *F* could be as high as 10^−5^ counts fs^−1^ or a flux of 10^20^ particles cm^−2^ s^−1^,^[^ ^50^ ^]^ and the value of *F* could reach 0.5 counts fs^−1^ in plasma FIBs.^[^ ^51^ ^]^ MC simulations at lower *F* values (e.g., F≤0.005) report discrete changes in the transient current from single events and simulate the SEE. This result suggests that our TDDFTB‐OS framework offers a theoretical tool to model device applications in space radiation conditions. At high fluxes (e.g., F≥0.5) under the ion beams, for example, the output signal is rather noisy and the correlation between irradiation events should be considered, which remains to be explored in future studies.

## 4 Conclusion

SPI processes are modeled in graphene electronics using RT‐TDDFT simulations extended to open electronic systems. This allows for the identification of outward propagation of irradiation‐induced damped electronic waves. The peak current is then calculated for devices to evaluate the potential for failure in the electric circuits. The signal is found to be mostly amplified at a moderate irradiation energy of 150 eV, which corresponds to delocalized plasmonic excitation. The excitation highly depends on the electronic and lattice structures, in contrast to the energy transfer in stopping power assessment, which is more sensitive to localized excitation and can be predicted from the properties of atoms. Based on the simulation results, a mapping of the dependence of the peak current on irradiation sites is generated, providing a database for device modeling under specific radiation conditions, such as those found in space environments. The response is shown to be determined by the electron density and ionic charges, which can be engineered through strain, defect, and phase changes that are particularly prominent in low‐dimensional materials.^[^ ^52^, ^53^, ^54^ ^]^ Although recent experimental studies clearly demonstrated the irradiation effects on the performance of nanoelectronics,^[^ ^6^ ^]^ resolving the underlying physics of SPI still remains challenging. Insufficient temporal and spatial resolutions limit the characterization of excited nuclear and electronic dynamics.^[^ ^5^, ^55^ ^]^ The roles of lattices and electronic structures thus cannot be addressed. The theoretical framework presented in the current work provides a unique tool to under the SPI effects at the first‐principles level and may guide future experimental designs.

## Conflict of Interest

The authors declare no conflict of interest.

## Author Contributions

Z.X. conceived the project. C.‐Y. developed the simulation code. Z.X. and C.‐Y. supervised the research. W.H. and L.Z. performed the research and contributed equally to this work. All authors participated in data analysis and paper writing.

## Supporting information

Supplementary Material

## Data Availability

The data that support the findings of this study are openly available in [Github] at https://github.com/xuzpgroup/LinxinZhai/tree/main/smallscience2023, reference number [20230325].
