# Supplementary material for: Current Transients in Graphene Electronics under Single‐Particle Irradiation
Source: Small Sci. 2023 Jun 11;3(7):2300011. doi: 10.1002/smsc.202300011 (PMC11935796; doi:10.1002/smsc.202300011)
Supplement: Supplementary file 1 — Supplementary Material [file SMSC-3-2300011-s001.pdf]

Supporting Information for Current Transients in Graphene Electronics under Single-Particle Irradiation

Wanzhen He[a,†], Linxin Zhai[a,†], Chi-Yung Yam[b,*], and Zhiping Xu[a,*]

[a] *Applied Mechanics Laboratory, Department of Engineering Mechanics, Tsinghua University, Beijing 100084, China*

[b] *Beijing Computational Science Research Center, ZPark II, Beijing 100193, China*

[†] *These authors contributed equally to this work.*

[*] *Email: xuzp@tsinghua.edu.cn, yamcy@csrc.ac.cn*

This Supporting Information Material includes

Supplementary Notes S1-S2

Supplementary Figures S1-S9

Supplementary References 1-7

Supplementary Notes S1-S2

S1. Details of charge analysis.

S2. The Monte Carlo algorithm.

Supplementary Note 1: Details of Charge Analysis

Time-dependent density functional tight-binding models extended to open systems (TDDFTB-OS) and Mülliken charge analysis in LODESTAR. The real-time time-dependent density functional tight-binding non-equilibrium Green function (RT-TDDFTB-NEGF) calculations coupled to the Ehrenfest dynamics were performed using our LODESTAR code. The DFTB parameters used are tested in combination for all O, N, C, H interactions[1]. Compared to ab-initio calculations, the reaction energies show errors below 1 kcal/mol. The geometry has an accuracy of 0.014 Å in the bond length and of ~ 2 degree in the bond angle.

Mülliken charge analysis (MCA) based on the electronic orbitals[2] is used to define the atomic charges in LODESTAR (**Figure S3**). This definition comes naturally in the tight-binding models and has been widely employed. The Bader charge is calculated from charge density, which requires numerical regeneration from the orbitals to the charge density in the tight-binding models and is less favored compared with MCA.

Real-time time-dependent density functional theory (RT-TDDFT) based calculations and Bader charge analysis in SIESTA. Our RT-TDDFT calculations in the closed-system approach are conducted for charge exchange and deposition analysis at different irradiation sites (**Figure 3d**) using SIESTA. The double-ζ polarized (DZP) orbitals are chosen as local basis sets. The adiabatic Perdew-Burke-Ernzerhof (PBE) parameterization of generalized gradient approximation (GGA) is used for the exchange-correlation functional[3], and the Troullier–Martins norm-conserving pseudopotentials for ion-electron interactions[4] without explicit consideration of the core electrons for

carbon. The cut-off energy for the basis set is 100 Ry, and the **k**-space is sampled by a $5 \times 5 \times 1$ Monkhorst-Pack grid[5]. Graphene is modeled by a $4 \times 4 \times 1$ supercell with a vacuum layer of 0.3 nm perpendicular to the basal plane of graphene for isolation. A single `H` atom is enforced with a normal velocity to simulate the single-particle irradiation (SPI) process, and its kinetic energy is 150 eV. The time steps for the electronic and nuclear dynamics are both 1 attosecond.

Bader charge analysis (BCA) based on the real-space charge density[6] is used to define the atomic charges in SIESTA. This definition prevails over the Mülliken charge since the latter is sensitive to the choice of basis sets. However, MCA can also be used if basis functions centered on atoms are chosen for the calculations of the electronic wavefunctions.

Summary In brief, MCA is based on electronic orbitals and widely used in the tight-binding methods as the generation of real-space charge density distribution from the information of the orbitals may lead to a loss in accuracy. BCA is conducted by using the real-space charge density and is thus robust for its minor dependence on the choice of basis sets.

Therefore, MCA is chosen for calculations of the atomic charges in LODESTAR, while the closed-system approach in SIESTA (**Figure 3d**) uses BCA. Both methods come to the same conclusion that charge exchange is more significant than charge deposition upon irradiation. The Mülliken charge exchange is $0.60e$, and the deposition is $0.23e$ for irradiation on the bond center with $K =$ 150 eV. Values of the Bader charge exchange/deposition are summarized in **Figure 3d**.

Supplementary Note 2: The Monte-Carlo Algorithm

A Monte Carlo (MC) algorithm is developed to simulate the transient current ($I(t)$) in the graphene devices under an irradiation flux F (**Figure 5**). The framework is based on a spatial sampling of the site-dependent responses of SPI events, following the contours of the peak current (**Figure 3a**). The signal measured from TDDFTB-OS simulations includes the steady-state current in absence of irradiation, $I_0 = 350$ nA, and a white noise below $0.02\% I_0$. SPI events are assumed to be independent, and correlated effects are ignored for practical consideration. The algorithm computes $I(t)$ as follows (**Figure S9**):

(1) Define the parameters, including the time step $\mathrm{d}t = 0.01$ fs, total time of simulation $T = 500$ fs, the average value of $I_0 = 350$ nA, and the damping time of the current under SPI $\tau_\mathrm{d} = 2$ fs.

(2) Define the irradiation flux F and read the map of site-dependent peak current from external sources of the TDDFTB-OS simulation results.

(3) At each MC time step, a uniformly-distributed random number r between 0 and 1 is generated. The irradiation event occurs if $r > F\mathrm{d}t$, and a second random number is then generated to sample the value of the induced peak current.

(4) Calculate the output signal at every timestep, $I(t) = I_\mathrm{s}(t) + I_\mathrm{i}(t)$. $I_\mathrm{s}(t)$ is generated by adding a white noise to the steady-state current, I_0, and $I_\mathrm{i}(t)$ is obtained from Step 3.

Supplementary Figures S1-S9

S1. Atomic structures of the simulation model containing a zigzag-edged graphene nanoribbon.

S2. Electronic excitation in graphene electronics under SPI.

S3. Initial and final charge states of the hydrogen projectile launched at different irradiation energies.

S4. Sampling of pristine and defective sites in graphene.

S5. Contours of the peak current on pristine and defective sites in graphene.

S6. Contours of peak current in pristine armchair-edged graphene nanoribbons (AGNRs).

S7. Contours of peak current on pristine and defective sites in 11-AGNRs.

S8. Contours of peak current in pristine graphene-nanoribbon devices.

S9. Flow chart of the Monte Carlo algorithm to simulate the transient current.

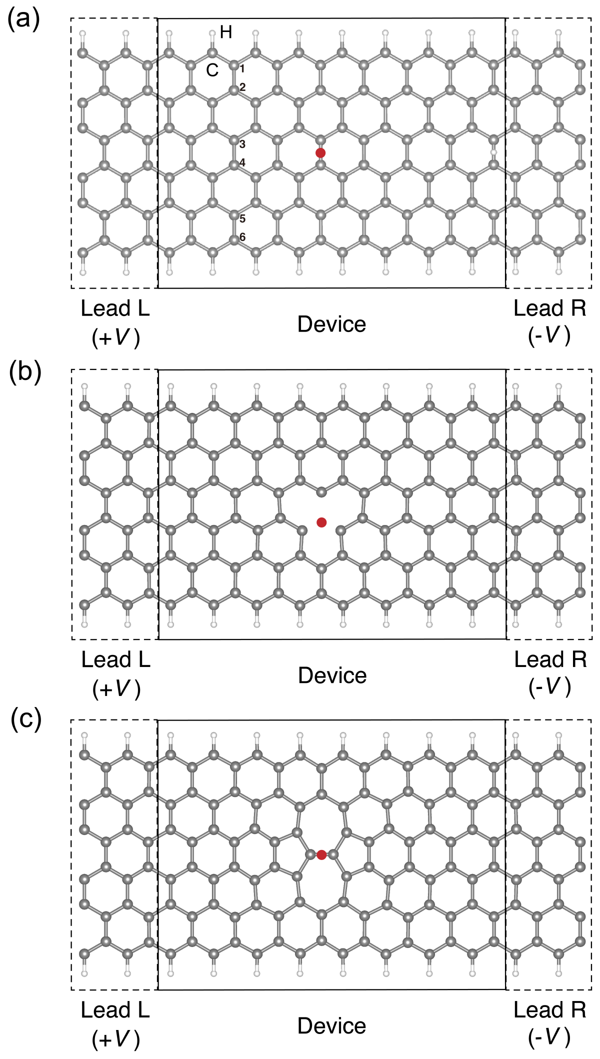

Figure S1 Atomic structures of the TDDFTB-OS simulation model containing a zigzag-edged graphene nanoribbon. Left (L) and right (R) leads are connected to the device (D) in an open system. A bias voltage V_b $(2V) = 0.1$ V is applied in all calculations. **Figure 2** is calculated for single-particle irradiation (SPI) on the bond center of the graphene lattice in (a), which is annotated by the red dot. (b) and (c) are the regions of single vacancy and Stone-Wales defects used in the calculation of **Figure 4**, respectively.

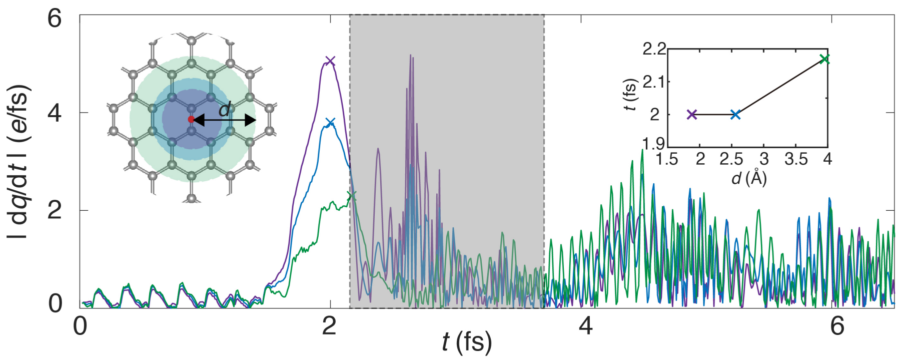

Figure S2 Electronic excitation in graphene electronics under SPI. Schematic illustration of charge density waves in a free-standing graphene device under SPI, from the focused ion beams (FIBs) or space radiation, for example. **b.** Changes in the atomic charge ($|\Delta q|$) in the area enclosed by a cut-off distance (d) from the site of single-H irradiation (the red dot). The results are plotted for the free-standing model, where the atoms in the device region are free to move. Compared to the constrained device (**Figure 1b**), the oscillation in amplitude is magnified in the freestanding model due to the coupling between electronic excitation and atomic displacement.

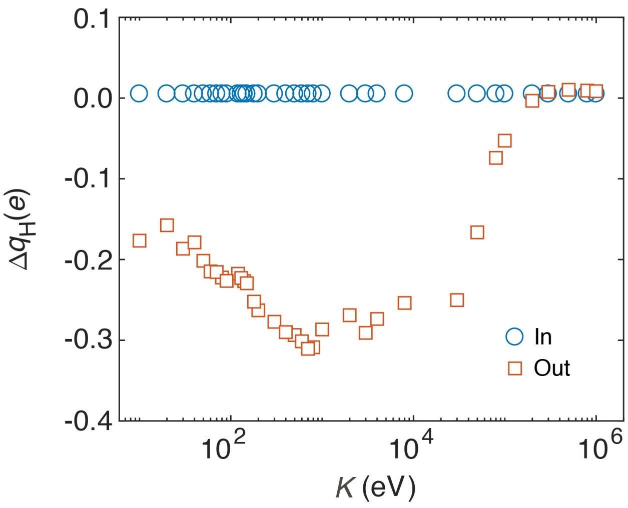

Figure S3 Initial and final charge states of the hydrogen projectile launched at different irradiation energies. Changes in the charge state of the H projectile before and after irradiation on graphene with the reference charge of atomic hydrogen. The atomic charge calculations are conducted at positions at least 0.5 nm away from the graphene to ensure convergence. The site of irradiation is the bond center, and the irradiation energy K ranges from 10 eV to 1 MeV. The results are plotted for the constrained model, where the atoms in the device region are fixed.

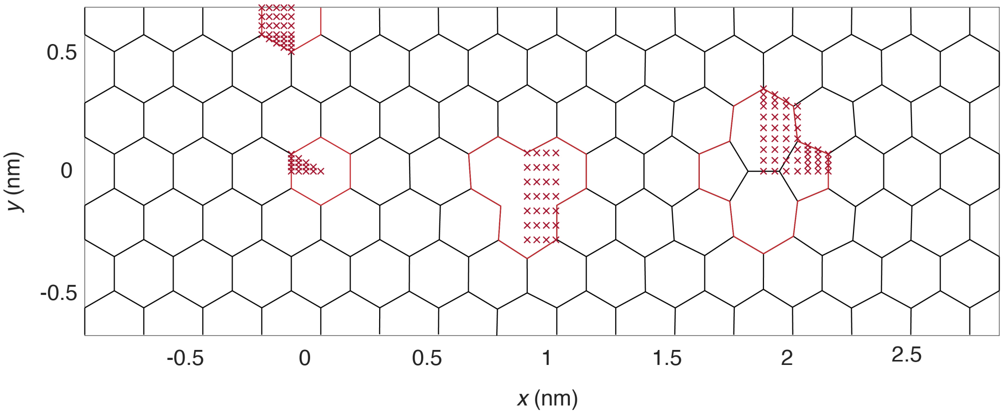

Figure S4 Sampling of pristine and defective sites in graphene. Sampled regions include edges, pristine regions, single vacancies, and Stone-Wales defects, from left to right. Irradiation sites in each region, denoted by red crosses, are chosen by following the lattice symmetry with 30, 15, 28, and 49 sampling points, respectively. The irradiation effects on defective structures are simulated separately, similar to the configuration illustrated in **Figure S1**, where the defects are located in the center of the device region. Defective structures are relaxed before irradiation.

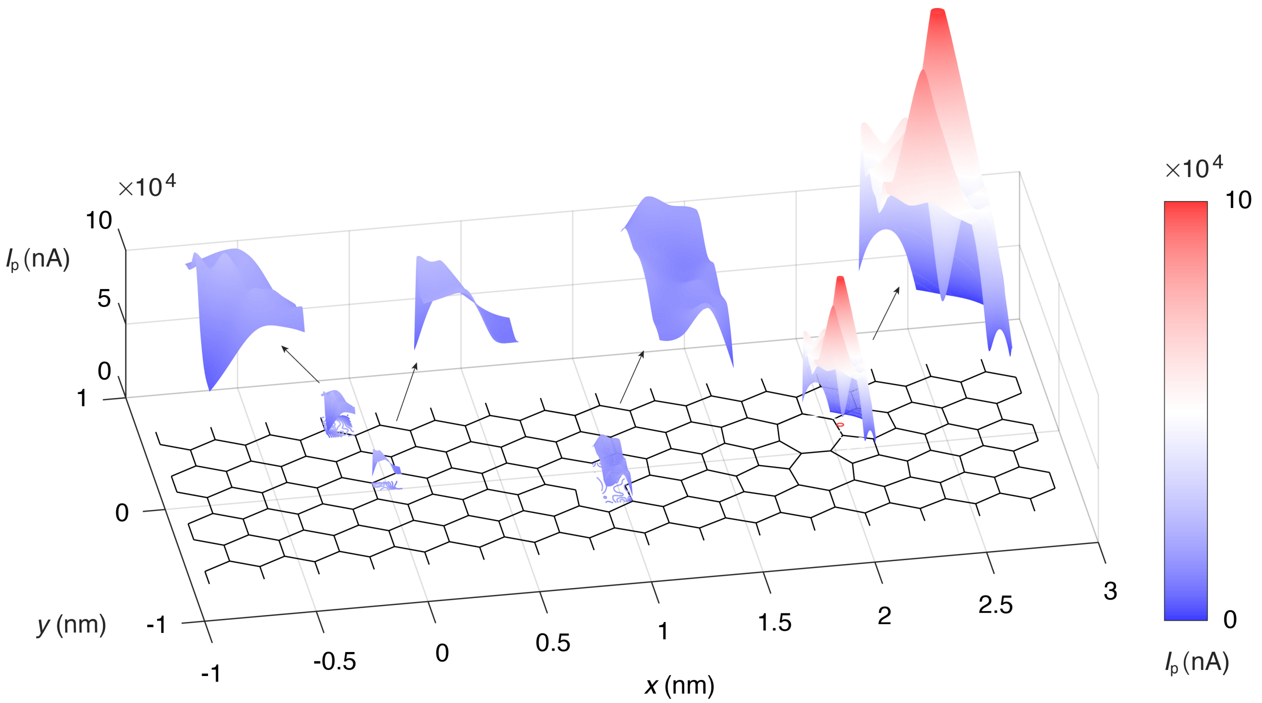

Figure S5 Contours of the peak current on pristine and defective sites in graphene. Contours of I_p in regions of the edges, pristine domains, single vacancies, and Stone-Wales defects (from left to right), interpolated from sampled sites in **Figure S4**. The irradiation energy K is 150 eV, and the bias voltage is $V_\mathrm{b} = 0.1$ V. The results are plotted for the freestanding model, where the atoms in the device region are free to move.

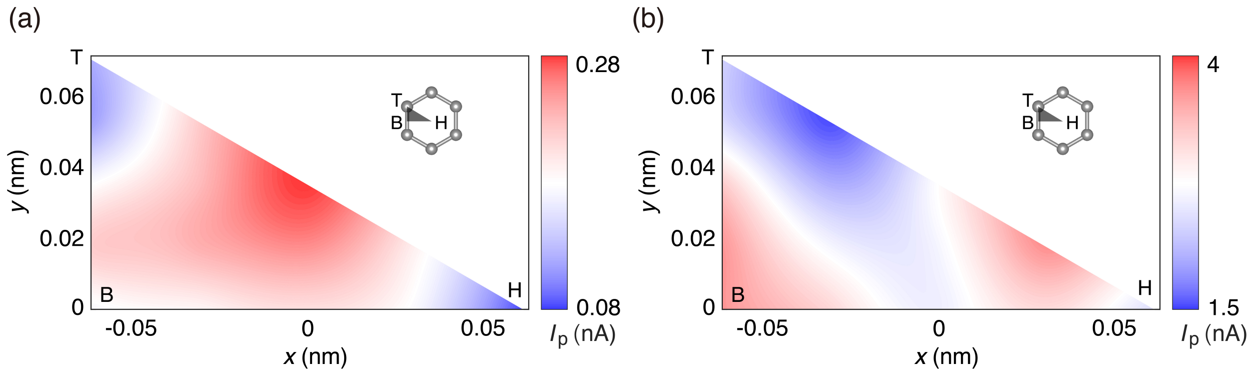

Figure S6 Contours of peak current in pristine armchair-edged graphene nanoribbons (AGNRs). 2D I_{p} map for irradiation sites sampled in a representative triangle of the graphene lattice, which is calculated at K is 150 eV and $V_{\mathrm{b}} = 0.1$ V. The results are plotted for the 13-AGNR (a) and 11-AGNR, respectively (b). The nomenclature of AGNRs follows Ref. [7]. In N-AGNR, there are N rows of carbon chains parallel to the ribbon edges. The 11-AGNR is metallic and the 13-AGNR is semiconducting with a band gap of 0.6 eV, as calculated by the DFTB model. The atoms in the device region are fixed.

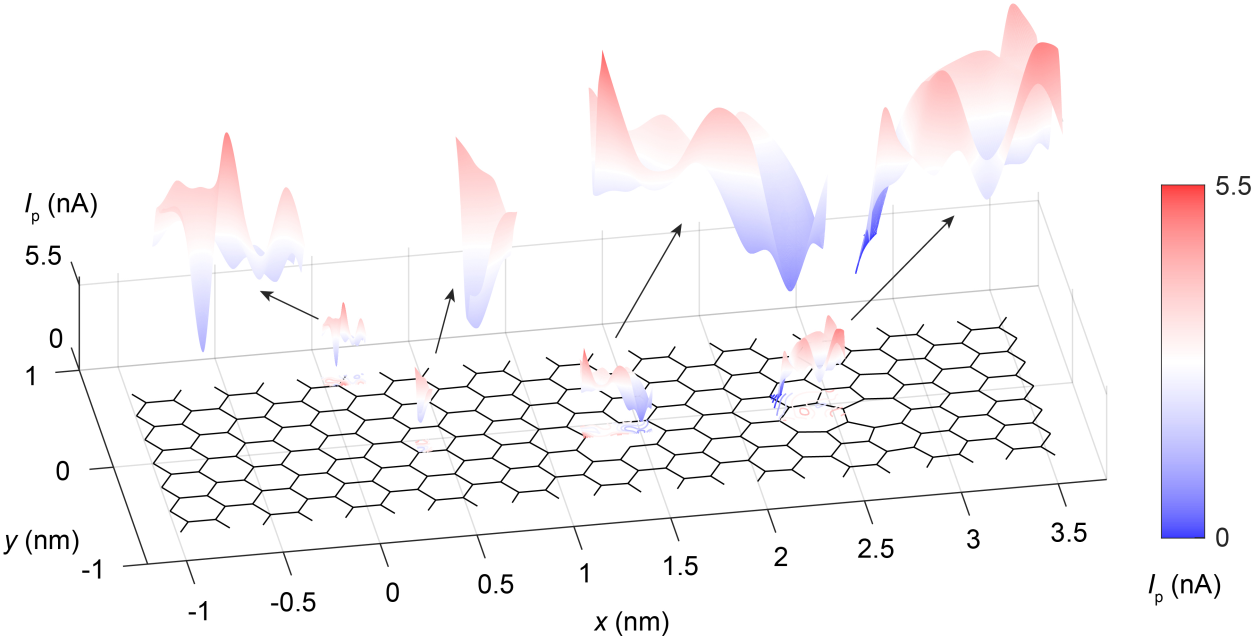

Figure S7 Contours of peak current on pristine and defective sites in 11-AGNRs. Contours of I_p in regions of the edges, pristine domains, single vacancies, and Stone-Wales defects (from left to right). The irradiation energy K is 150 eV, and the bias voltage is $V_\mathrm{b} = 0.1$ V. The atoms in the device region are fixed.

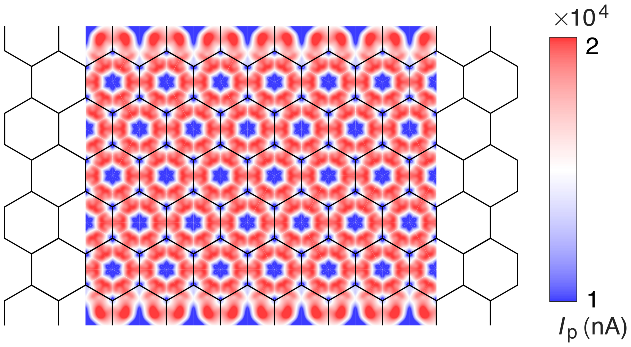

Figure S8 Contours of peak current in pristine graphene-nanoribbon devices. The plot is extended from calculations by spatially sampling the pristine and edge regions in **Figure S3**. The atoms in the device region are fixed.

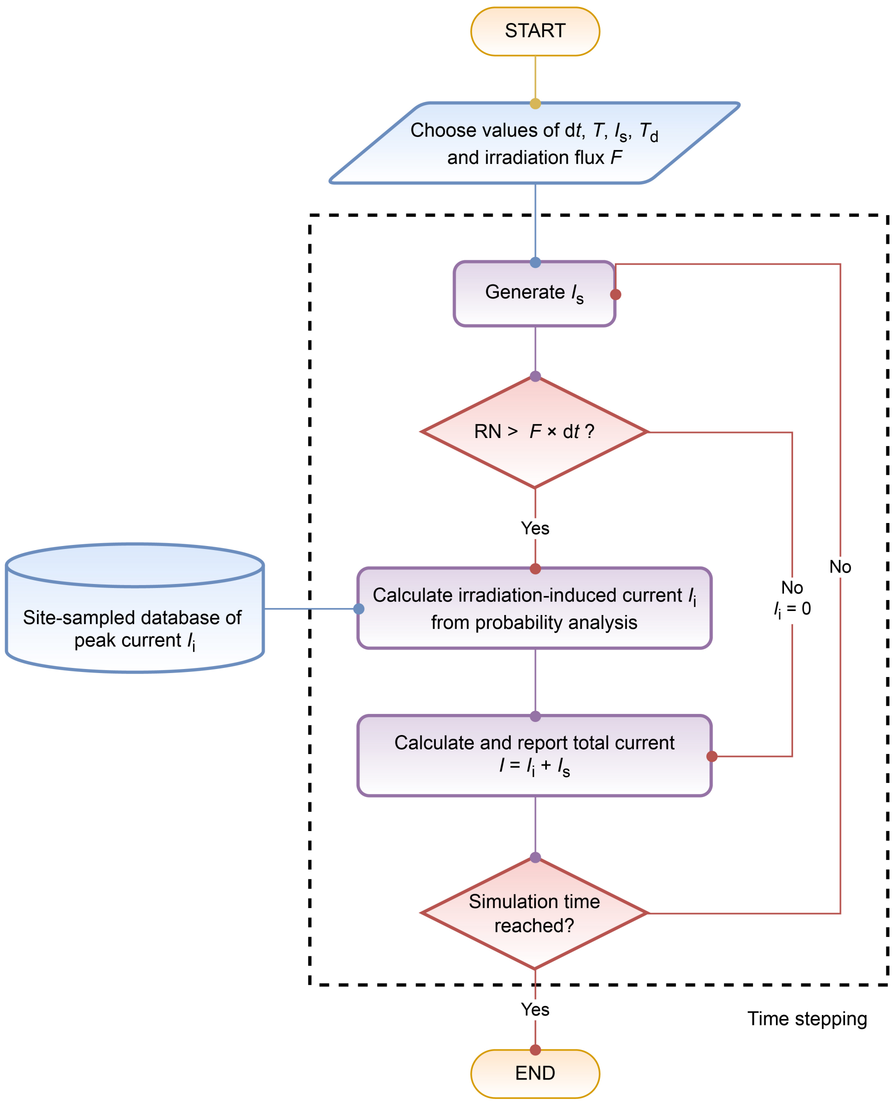

Figure S9 Flow chart of the Monte Carlo algorithm to simulate the transient current. RN is the uniformly-distributed random number generated between 0 and 1. Other parameters are explained in **Supplementary Note 2**. The red dashed box marks the Monte-Carlo steps, and the blue ones indicate the inputs.

Supplementary References

1. M. Elstner, D. Porezag, G. Jungnickel, J. Elsner, M. Haugk, T. Frauenheim, S. Suhai, G. Seifert, *Phys. Rev. B* **1998**, *58*, 11 7260.

2. F. Martin, H. Zipse, *J. Comput. Chem.* **2005**, *26*, 1 97.

3. J. P. Perdew, K. Burke, M. Ernzerhof, *Phys. Rev. Lett.* **1996**, *77*, 18 3865.

4. N. Troullier, J. L. Martins, *Phys. Rev. B* **1991**, *43*, 3 1993.

5. H. J. Monkhorst, J. D. Pack, *Phys. Rev. B* **1976**, *13*, 12 5188.

6. E. Sanville, S. D. Kenny, R. Smith, G. Henkelman, *J. Comp. Chem.* **2007**, *28*, 5 899.

7. Y.-W. Son, M. L. Cohen, S. G. Louie, *Phys. Rev. Lett.* **2006**, *97*, 21 216803.
